# Tracking a refined eIF4E-binding motif reveals Angel1 as a new partner of eIF4E

**DOI:** 10.1093/nar/gkt569

**Published:** 2013-06-27

**Authors:** Pauline Gosselin, Yvan Martineau, Julia Morales, Mirjam Czjzek, Virginie Glippa, Isabelle Gauffeny, Emmanuelle Morin, Gildas Le Corguillé, Stephane Pyronnet, Patrick Cormier, Bertrand Cosson

**Affiliations:** ^1^UPMC Univ Paris 06, UMR 7150, Mer et Santé, Station Biologique, F-29680 Roscoff, France, ^2^CNRS, UMR 7150, Mer et Santé, Station Biologique, F-29680 Roscoff, France. ^3^Université Européenne de Bretagne, Bretagne, Roscoff, France, ^4^INSERM, UMR 1037, Centre de Recherche en Cancérologie de Toulouse, Toulouse 31432, France, ^5^UPMC Univ Paris 06, UMR 7139, Végétaux Marins et Biomolécules, Station Biologique, F-29680 Roscoff, France, ^6^CNRS, UMR 7139, Végétaux Marins et Biomolécules, Station Biologique, F-29680 Roscoff, France, ^7^UPMC Univ Paris 06, FR2424, ABiMS, Station Biologique, F-29680 Roscoff, France and ^8^CNRS, FR2424, ABiMS, Station Biologique, F-29680 Roscoff, France

## Abstract

The initiation factor 4E (eIF4E) is implicated in most of the crucial steps of the mRNA life cycle and is recognized as a pivotal protein in gene regulation. Many of these roles are mediated by its interaction with specific proteins generally known as eIF4E-interacting partners (4E-IPs), such as eIF4G and 4E-BP. To screen for new 4E-IPs, we developed a novel approach based on structural, *in silico* and biochemical analyses. We identified the protein Angel1, a member of the CCR4 deadenylase family. Immunoprecipitation experiments provided evidence that Angel1 is able to interact *in vitro* and *in vivo* with eIF4E. Point mutation variants of Angel1 demonstrated that the interaction of Angel1 with eIF4E is mediated through a consensus eIF4E-binding motif. Immunofluorescence and cell fractionation experiments showed that Angel1 is confined to the endoplasmic reticulum and Golgi apparatus, where it partially co-localizes with eIF4E and eIF4G, but not with 4E-BP. Furthermore, manipulating Angel1 levels in living cells had no effect on global translation rates, suggesting that the protein has a more specific function. Taken together, our results illustrate that we developed a powerful method for identifying new eIF4E partners and open new perspectives for understanding eIF4E-specific regulation.

## INTRODUCTION

The control of gene expression at the mRNA level is a complex process that is critical during many physiological events such as cell cycle, cell growth, differentiation, aging and cell death. In eukaryotes, the eukaryotic initiation factor 4E (eIF4E) plays essential roles at several steps of the mRNA life cycle: translation initiation, nuclear export [reviewed in (1)], cytoplasmic localization and stability control (2). The deregulation of eIF4E activities is a key component in cancer initiation and progression (3,4). Controlling eIF4E functions is therefore a crucial step in normal cell proliferation and survival.

During translation initiation, eIF4E binds the cap structure of mRNA and recruits eIF4G, a large scaffolding protein that acts as a docking site for several proteins required for bridging the ribosome and the mRNA (5,6). The interaction between eIF4E and eIF4G is inhibited in a competitive manner by the small translational repressor 4E-BP, which shares a consensus eIF4E-binding motif YxxxxLΦ (where x is a variable amino acid and Φ is a hydrophobic residue) with eIF4G (7). The motif-containing central peptide of 4E-BP (corresponding to residues 51–67 of human 4E-BP1) acts as a molecular mimic of eIF4G on the convex dorsal surface of eIF4E, forming an L-shaped structure with an extended chain region and a short α-helix (8). Nevertheless, the interaction with eIF4E does not depend only on the central peptide of 4E-BP as currently thought. In fact, the binding footprint of 4E-BP appears to be larger and involves fuzzy contacts between 4E-BP extremities and the eIF4E surface (9).

In the nucleus, eIF4E promotes nucleocytoplasmic transport of a selected subset of mRNAs. These transcripts, such as cyclin D1 and ODC, are involved in cell cycle regulation (1,10,11) and carry a specific 4E-sensitivity element in their 3′UTR (12). Several key regulators of eIF4E-dependent mRNA export have been identified, most of them containing the consensus eIF4E-binding motif found in 4E-BP or eIF4G (13–15).

Beyond well-known regulators of mRNA export and translation initiation, some other eIF4E-interacting partners (4E-IPs) have been discovered (16). These 4E-IPs, such as Maskin, Bicoid, DDX3, 4E-T, Gemin5 and GIGYF2, play fundamental roles in cell cycle progression, metabolism, development, tumor formation and responses to various stimuli (2,17–21). Consequently, finding novel interacting partners of eIF4E would help to understand cellular mechanisms controlled by eIF4E activity.

In the present study, we used a new approach based on structural and *in silico* analyses to find new 4E-IPs. Using a refined eIF4E-binding motif to search databases for potential 4E-IPs, we found a CCR4 family member, Angel1, which displays an eIF4E-binding motif in its C-terminal domain.

## MATERIALS AND METHODS

### Plasmids

Total RNA from 293 cells was prepared using Trizol purification, and reverse transcribed using the Superscript reverse transcriptase (Invitrogen) according to the manufacturer’s instructions. Angel1 cDNA was amplified and subcloned in different vectors as indicated in Supplementary Methods.

### Antibodies

The antibodies used for western blotting and immunofluorescence are detailed in Supplementary Methods.

### Phylogeny

The original alignment produced by T-Coffee (22)/M-Coffee (23) on 970 sites was optimized using trimAl (24). PhyML (25) was used to reconstruct a maximum likelihood (ML) phylogenetic tree (performed with the LG substitution model (26), 1000 bootstraps and 4 substitution rate categories). Sequences used are detailed in Supplementary Methods.

### Cell lines

Growth conditions and Angel1-shRNA accession numbers are detailed in Supplementary Methods.

### Immunoprecipitation and m^7^GTP purification

Details are given in Supplementary Methods.

### Expression, production, eIF4E-binding assay

The wild-type and mutant proteins GST-A1 and GST-A1YA were overexpressed in *E. coli* [Rosetta (BL21), Novagen] and purified on a glutathione sepharose 4B column (Amersham Pharmacia Biotech) according to the manufacturer’s instructions. The eIF4E-binding assay and immunoblot analysis are described in Supplementary Methods.

### Immunofluorescence

Images were collected on a confocal Leica SP5 microscope using a 40× or 63× oil objective. Cells were prepared as described in Supplementary Methods.

### Cell fractionation

For cell fractionation, the protocol is derived from Culjkovic-Kraljacic *et al.* (27). Briefly, around 2.10^7^cells were washed twice in ice-cold PBS and collected (1000*g* for 3 min). Then cells were resuspended with slow pipetting in 500 µl of hypotonic lysis buffer (10 mM Tris pH8.0, 1.5 mM MgCl_2_, 10 mM NaCl, 1 mM DTT) and vortexed for 4 s. After centrifugation at 1000*g* (4°C) for 3 min, supernatant (cytoplasmic fraction) was transferred into a fresh tube. Pellet fraction was washed with hypotonic lysis buffer and resuspended in 500 µl lysis buffer A (10 mM Tris pH 8.0, 140 mM NaCl, 1.5 mM MgCl_2_, 0.5% NP40, 1 mM DTT). After centrifugation at 1000*g* (4°C) for 3 min, supernatant (microsomal fraction) was transferred into a fresh tube. Pellet-nuclear fraction was washed once and resuspended in 500 µl of lysis buffer A, transferred to a 5-ml round-bottom tube and 1/10 volume (50 µl) of detergent stock [3.3% (w/v) sodium deoxycholate, 6.6% (v/v) Tween 40, in DEPC H20] was added under slow vortexing (this prevents nuclei from clumping) and incubated on ice for 5 min. This suspension was transferred back to a 1.5 ml eppendorf and spun at 1000*g* at 4°C for 3 min. Supernatant-postnuclear fraction was transferred to a fresh tube. The pellet-nuclear fraction was washed with buffer A and resuspended in 500 µl of lysis buffer A supplemented with 0.1% SDS and sonicated.

## RESULTS

### Angel1, a member of the CCR4 family, acquired an eIF4E-binding motif in vertebrates

**Figure 1.**
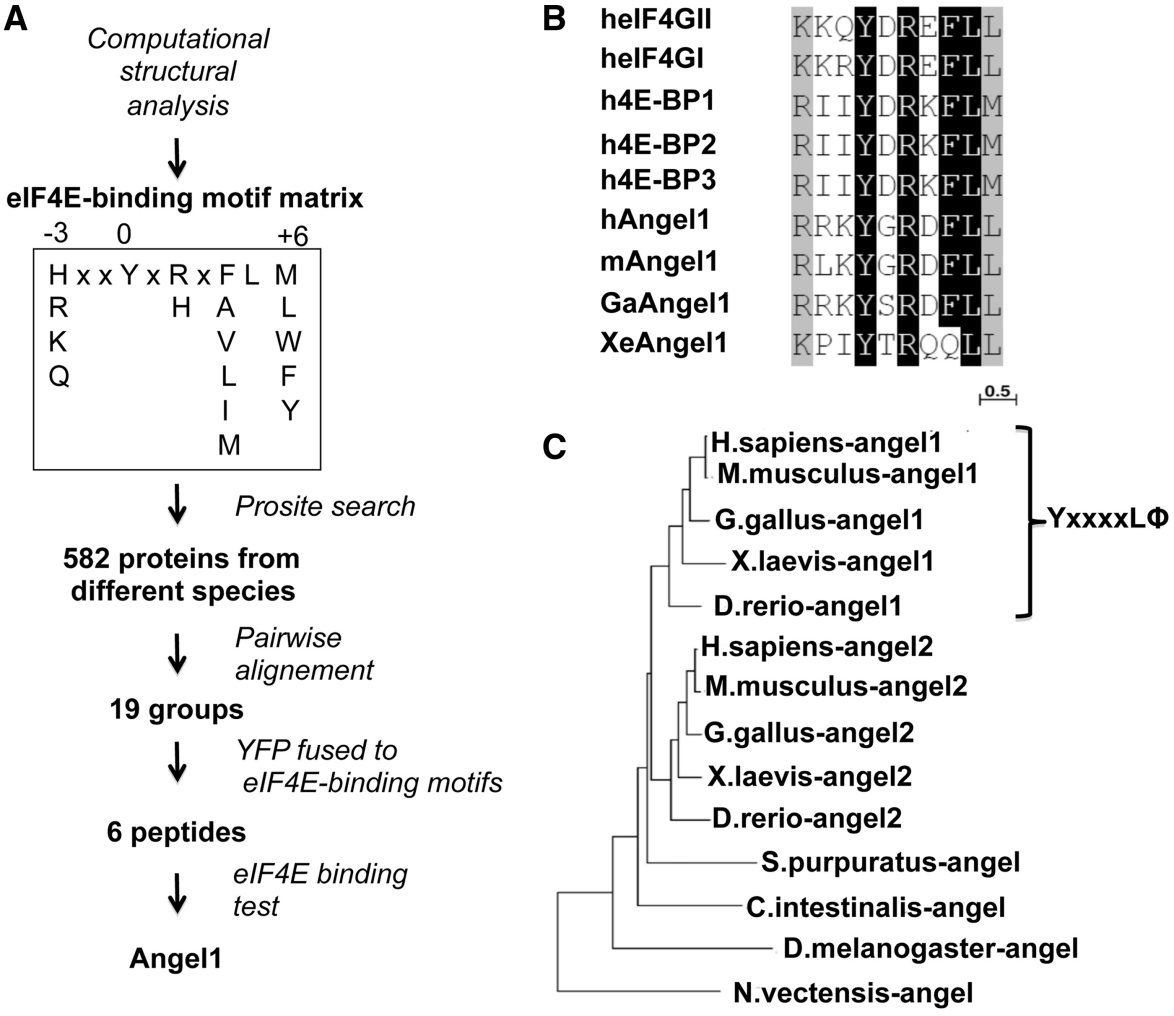
New screening reveals a novel 4E-IP, Angel1, which acquired an eIF4E-binding motif in vertebrates. (**A**) Combination of structural, *in silico* and m^7^GTP chromatography approaches reveal that Angel1 is a novel eIF4E-interacting protein. See text for details. (**B**) The putative eIF4E-binding motifs of Angel1 mouse (m), human (h), chicken (Ga) and Xenopus (Xe) were aligned over several eIF4E-binding proteins. Residues that were identical or conserved in >75% of the sequences are shaded in black and gray, respectively. The seven last amino acids correspond to the consensus eIF4E-binding motif YxxxxLΦ. (**C**) Unrooted phylogenetic tree of Angel-related sequences in 9 species. The presented tree was constructed using the Maximum Likelihood method (see SI). The brace indicates the sequences that contain the consensus motif (YxxxxLΦ).

To find new eIF4E partners, our first approach was to look for sequences in protein databases that contained an eIF4E-binding consensus motif, YxxxxLΦ. However, scanning databases with the webserver Prosite (http://www.expasy.org/tools/scanprosite) did not produce any significant hits because the probability of finding this consensus motif randomly in databases is too high. To define a more accurate consensus sequence, we performed a structural analysis using the crystal structures of the complexes formed between eIF4E and peptides derived from well-known 4E-IPs: 4E-BP1 and eIF4GI [PDB ID: 1EJ4 and 1EJH, (8)]. We evaluated the change in the 3D structure after substituting each residue of the peptides from positions −3 to +6 (annotated from the conserved tyrosine of the consensus motif) using Turbo-Frodo software (28). The resulting set of sequences that were tolerated by the peptides and that could still interact with eIF4E constituted the refined eIF4E-binding motif matrix (Figure 1A). Scanning the multi-species UniProtKb/Swiss-Prot database with Prosite revealed that the refined eIF4E-binding motif was present in 582 sequences (out of 5.10^5^ protein sequences in the database, data not shown). As expected, we found orthologs of already known eIF4E-interacting proteins, such as eIF4Gs or 4E-BPs. Because functionally relevant sequences are expected to be conserved throughout evolution, orthologs that share the refined eIF4E-binding motif therefore have a greater probability of effectively binding eIF4E. By pairwise alignment of the 582 sequences containing the consensus motif, we found 19 groups of at least three orthologs that shared a coverage rate of >75% of their lengths and a percentage of identity >50%. Among these groups, 14 groups had not been previously characterized as binding partners of eIF4E (Supplementary Figure S1). To validate the functionality of each putative eIF4E-binding motif, we fused the human homolog motif of each group to yellow fluorescent protein (YFP) as carrier. Six of the 14 fusion proteins were successfully produced in rabbit reticulocyte lysate in presence of ^35^S methionine before being transferred on an m^7^GTP chromatography column pre-loaded with GST-eIF4E. As expected, YFP fused to the eIF4GI motif was retained on the eIF4E column (Supplementary Figure S2, lane 11). Interestingly, the YFP fused to the putative eIF4E-binding motif taken from a protein identified as Angel1 was also retained on the eIF4E column (Supplementary Figure S2, lane 15). Furthermore, the full-length Angel1 protein that we produced in rabbit reticulocyte lysate also bound to the eIF4E column (Supplementary Figure S3, lane 6). The Angel1 motif showed high similarity with the YxxxxLΦ motif in eIF4G and 4E-BP (Figure 1B). The replacement of the conserved tyrosine with an alanine in the eIF4G motif prevented binding to eIF4E (7). Importantly, we also observed that this mutation in the motif of Angel1 abolished the interaction with eIF4E (Supplementary Figure S3, lane 8). Never before mentioned as a potential 4E-IP, Angel1 has been described as a member of the CCR4 family due to its conserved C-terminal endo-exonuclease-phosphatase domain (EEP) found in CCR4 or Nocturnin proteins (Supplementary Figure S4) (29,30). The Angel1 locus comes from a duplication that occurred immediately prior to or early in vertebrate divergence (31), strongly suggesting that, through genome duplication in vertebrates, the ancestral gene coding for the Angel protein produced two paralogs coding for Angel1 and Angel2 (Figure 1C). Interestingly, we observed that the eIF4E-binding motif is only found in the C-terminal domain of Angel1 sequences (Figure 1C and Supplementary Figure S4). Therefore, we assumed that the eIF4E-binding motif in the Angel1 sequence appeared simultaneously with or immediately after the duplication of the ancestral gene *angel* and has been conserved throughout the evolution of vertebrates, suggesting that it has functional relevance.

### Angel1 is an eIF4E-interacting protein

**Figure 2.**
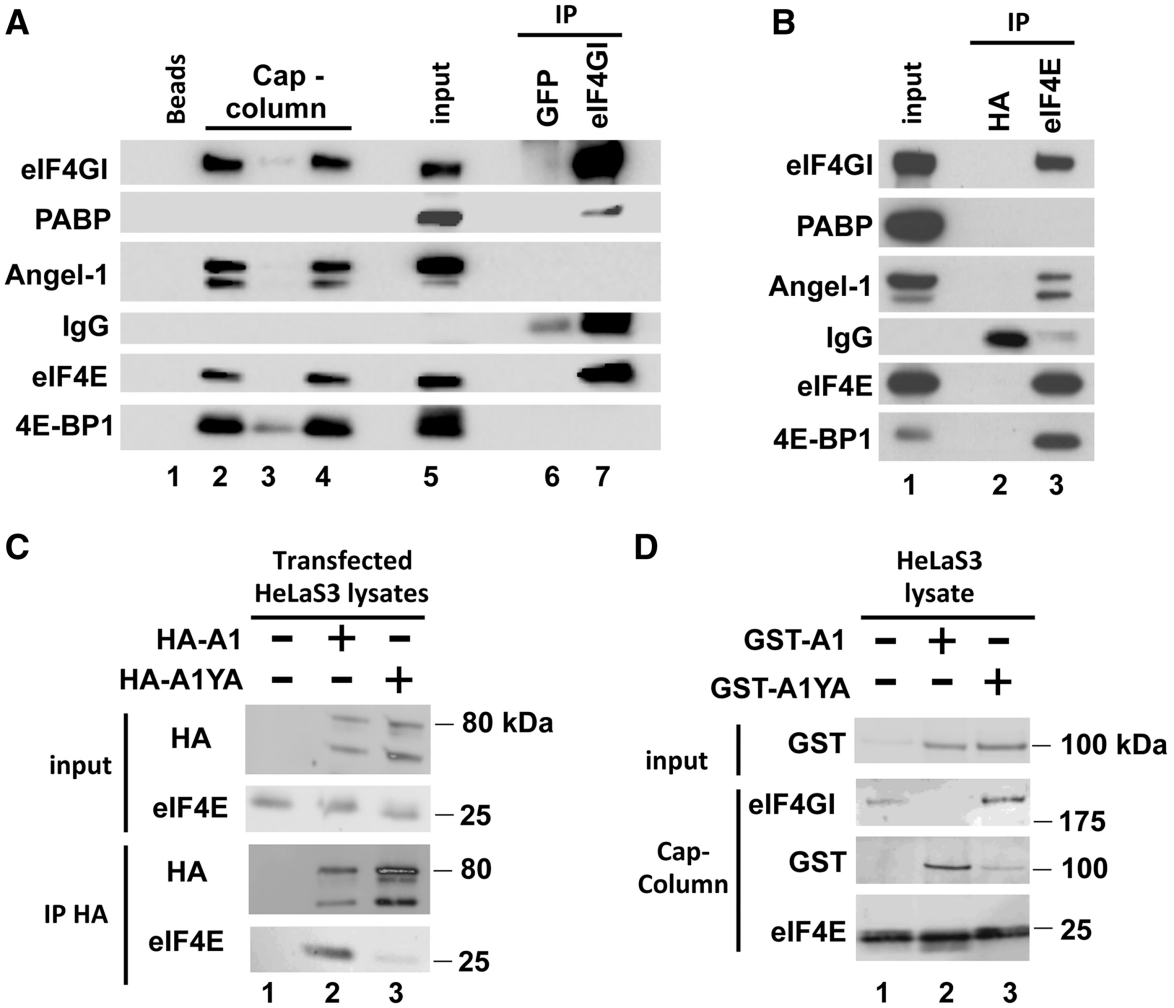
Angel1 interacts with eIF4E through its eIF4E-binding motif. (**A, B**) One HeLa S3 cell extract supplemented with RNase A was incubated with m^7^GTP beads or used to perform immunoprecipitation with indicated antibodies. Bound proteins were analyzed by western blotting. (A) Sepharose beads or m^7^GTP beads (cap-column assay) were incubated directly with Laemmli buffer (lanes 1 and 2). m^7^GTP beads were incubated with 200 µM free m^7^GTP; eluates (lane 4) and residual proteins attached to m^7^GTP beads (lane 3) were resolved on SDS-PAGE. Immunoprecipitations with anti-eIF4GI (lane 7) or isotype control antibody (anti-GFP, lane 6) contain immunoglobulin (IgG). Total lysate is presented in lane 5. (B) Immunoprecipitation using anti-eIF4E (lane 3) or isotype control antibody (anti-HA, lane 2) were performed with the same total lysate (lane 1) as in A. (**C**) Angel1 interacts with eIF4E through the conserved eIF4E-binding sequence. HeLa S3 cells were mock-transfected, or transfected with HA-A1 or HA-A1YA expressing vectors. Cell lysates were subjected to HA-immunoprecipitation. Whole lysates (input) and immnoprecipitates (IP HA) were analyzed by immunoblotting. (**D**) Angel1 competes with eIF4GI for binding to eIF4E *in vitro*. HeLa S3 cell lysates were supplemented with wild-type (lane 2) or mutant Angel1 (lane 3) GST-fusion protein (1 µg each) and incubated with m^7^GTP beads. 1/50 of total extracts (Input) and proteins bound to m^7^GTP beads (cap-column) were analyzed by western blot. The ability of recombinant wild-type or mutant Angel1 to bind endogenous eIF4E and displace eIF4GI was monitored using an anti-GST antibody.

To analyze the association between cellular Angel1 and eIF4E, HeLa S3 cell extracts underwent affinity chromatography on m^7^GTP-sepharose beads (Figure 2A). To exclude the possibility of RNA-mediated interactions, a cap-column assay was performed in the presence of RNase A. In our western blot experiments, Angel1 generally appeared as a doublet, probably owing to a post-transcriptional process that remains to be identified. The endogenous Angel1 protein was retained on the m^7^GTP column (Figure 2A, lane 2), as well as the eIF4E partner eIF4GI. In presence of free m^7^GTP, Angel1 was eluted together with eIF4E (Figure 2A, lane 4) and was not retained on m7-GTP beads (Figure 2A, lane 3). Noting that Angel1, eIF4GI and 4E-BP1 all have a similar eIF4E-binding motif, we performed eIF4GI immunoprecipitation using the same RNase-treated extract. As expected, we found that PABP and eIF4E were co-immunoprecipitated with eIF4GI, whereas 4E-BP1 and Angel1 did not (Figure 2A, lane 7). To further confirm the existence of the eIF4E/Angel1 complex in cells and to exclude any possible direct interaction of Angel1 with the cap structure, we performed eIF4E immunoprecipitation using the same RNase-treated extract. Angel1 co-precipitated with eIF4E, as well as eIF4GI and 4E-BP1, while PABP did not (Figure 2B, lane 3). These three eIF4E-binding partners therefore use the same binding motif, suggesting that endogenous Angel1 associates with a complex containing eIF4E through a direct protein–protein interaction. Altogether, these results indicate the existence of three mutually exclusive complexes, eIF4GI/eIF4E, 4E-BP1/eIF4E and Angel1/eIF4E.

It was then important to assess the function of the potential eIF4E-binding motif YxxxxLΦ in the Angel1 sequence (Figure 2C). In HeLa S3 cells we expressed hemagglutinin (HA)-tagged Angel1 and its variant A1YA, in which the conserved Tyr_506_ in the putative eIF4E-binding site was replaced by an alanine. We first checked by western blot that the HA-A1 and HA-A1YA proteins were similarly expressed following transfection of HeLa S3 cells (Figure 2C, Input, lanes 2 and 3). HA-A1 and HA-A1YA expressed in cells had two major forms with sizes of 80 and 74 kDa (Figure 2C, lanes 2, 3), suggesting again that Angel1 is post-translationally modified. Because the exogenous Angel1 protein is HA-tagged at the N-terminus, one possibility is a C-terminal truncation of the protein. Performing anti-HA immunoprecipitation, HA-A1 brought down eIF4E (Figure 2C, lane 2), while HA-A1YA did not (Figure 2C, lane 3), demonstrating that the eIF4E-binding site of Angel1 is required for its association with eIF4E.

Angel1 and eIF4GI share the same consensus eIF4E-binding motif; we therefore expected that Angel1 was able to displace the eIF4G/eIF4E interaction. GST-A1 and GST-A1YA proteins were produced in bacteria and purified on glutathione sepharose. Recombinant proteins were incubated with HeLa cell extracts, and endogenous eIF4E was purified on an m^7^GTP column. Proteins associated with eIF4E were analyzed by western blotting (Figure 2D). GST-A1 associated with endogenous eIF4E, but GST-A1YA did so only weakly (Figure 2D, compare lanes 2 and 3). In particular, binding of GST-A1 to endogenous eIF4E impaired the interaction between eIF4E and endogenous eIF4GI (Figure 2D, compare lanes 2 and 3). These results demonstrate that, in cell extracts, recombinant Angel1 competes with eIF4GI for binding to eIF4E.

**Figure 3.**
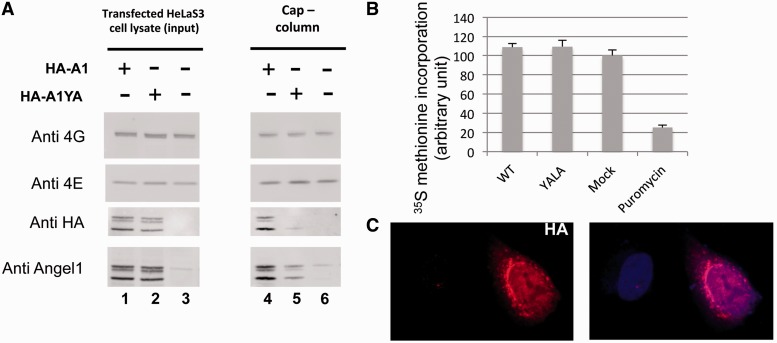
Overexpressed Angel1 neither competes with eIF4G nor affects general translation activity. HeLa S3 cells were mock-transfected, or transfected with HA-A1 or HA-A1YA expressing vectors. (**A**) Transfected cells were lysed and used for m^7^GTP chromatography and analyzed by immunoblotting. The membrane incubated with the anti-HA tag was then reprobed with the anti-Angel1 antibody. (**B**) In parallel, 24 h after transfection, cells were incubated with ^35^S-methionine and treated as described in ref. 56 using TCA to precipitate labeled proteins. ^35^S-methionine incorporation into proteins is expressed as a percentage of the mock-transfected control (*n* = 5). (**C**) Localization of HA-Angel1 was determined by indirect immunofluorescence with anti-HA and anti-Rat IgG-TRITC antibodies. Nuclei were stained with 1 µg/ml of Hoescht.

We next addressed whether Angel1 could also compete *in vivo* with eIF4G. HA-A1 and HA-A1YA were expressed in HeLa S3 cells and eIF4GI bound to eIF4E was monitored using a cap-column assay (Figure 3A). As expected, only HA-A1 bound efficiently to eIF4E, while HA-A1 and HA-A1YA were expressed at similar levels. However, HA-A1 did not alter the *in vivo* eIF4GI interaction with eIF4E (Figure 3A, compare lanes 1 and 2 with lanes 4 and 5).

Because eIF4E availability for eIF4G appears to be independent of Angel1, we hypothesized that manipulating Angel1 levels in living cells would have only a marginal effect on global translation rates. To test this hypothesis, Angel1 was overexpressed and protein synthesis was measured by ^35^S-methionine incorporation (Figure 3B). As expected, Angel1 overexpression did not affect the global protein synthesis rate. It is interesting to note that the overexpressed protein is localized in the perinuclear area (Figure 3C). Furthermore, polysome profiles in Sh-Angel1-expressing cell lines were not affected (Supplementary Figure S5C).

**Figure 4.**
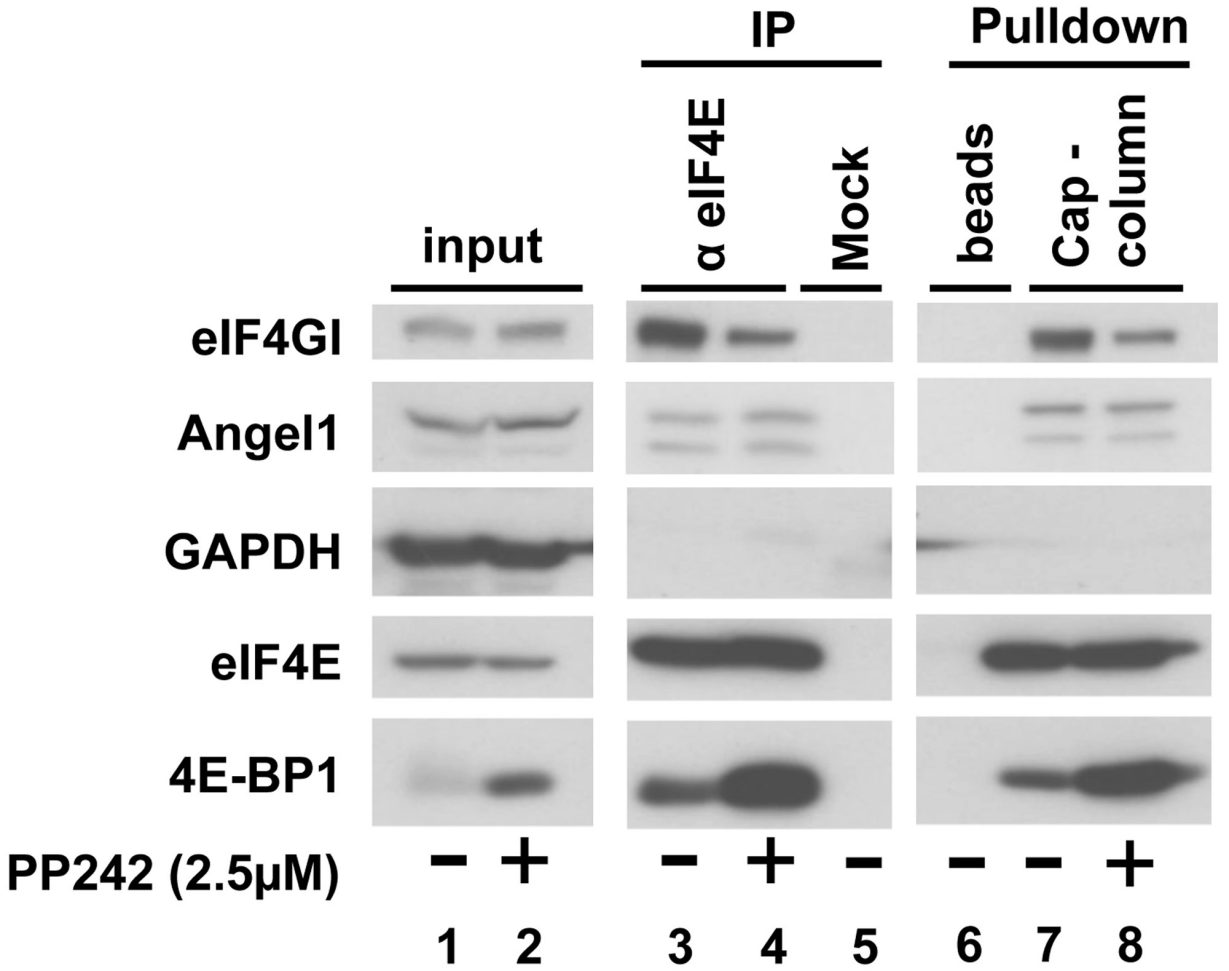
Angel1–eIF4E interaction is not sensitive to mTOR inhibition. HeLa S3 cells were treated with or without 2.5 µM PP242 (mTOR inhibitor) for 1 h. Cell extracts were incubated with m^7^GTP beads (cap-column, lanes 7 and 8), α-eIF4E-sepharose beads (lanes 3 and 4) or sepharose beads alone (lanes 5 and 6), as described in Figure 2A and B. Whole cell lysates (lanes 1 and 2) and bound proteins were analyzed by immunoblotting.

It is well known that hypophosphorylated 4E-BP1 competes with eIF4GI for a common binding site on eIF4E (32); we therefore explored whether hypophosphorylated 4E-BP1 could disrupt the Angel1–eIF4E interaction in living cells (Figure 4A). To induce *in vivo* 4E-BP1 hypophosphorylation, HeLa S3 cells were treated with PP242 (Figure 4A, lanes 2, 4, 8), an active-site mTOR inhibitor (33). Proteins from untreated and PP242-treated HeLa S3 cell extracts were immunoprecipitated using an anti-eIF4E antibody (Figure 4A, respectively lanes 3 and 4) or purified on a cap-column (Figure 4A, lanes 7 and 8). As expected, the amount of 4E-BP1 bound to eIF4E increased significantly after PP242 treatment (Figure 4A, compare lanes 3 and 4; lanes 7 and 8, bottom panels) while the quantity of eIF4GI bound to eIF4E decreased (Figure 4A, top panels). Strikingly, the Angel1 association with eIF4E was not affected when cells were treated with PP242. Taken together these data demonstrate that the formation of the Angel1/eIF4E complex is independent of the mTOR signaling pathway and its downstream target 4E-BP.

Therefore, our results demonstrate that Angel1 associates *in vivo* with eIF4E. However, eIF4E availability for eIF4G or 4E-BP—and consequently the global protein synthesis rate—are independent of this new eIF4E partner.

### Angel1 is predominantly found in the cytoplasmic perinuclear area and co-localizes with eIF4E in small particles

**Figure 5.**
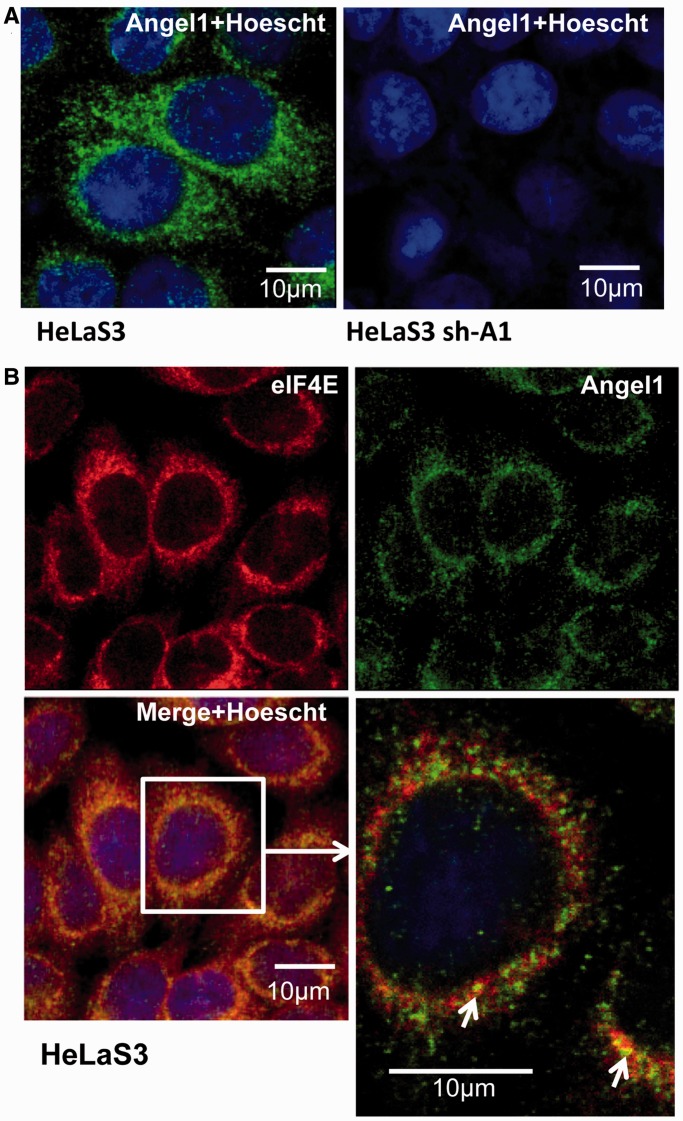
Angel1 co-localizes specifically with eIF4E in small perinuclear granules. (**A**) Immunofluorescence staining was performed with the anti-Angel1 antibody (Sigma) and an Alexa 488-conjugated anti-rabbit secondary antibody (green) on HeLa S3 cells or sh-Angel1 expressing cell lines. Nuclei were stained with 1 µg/ml of Hoescht. The subcellular localization of Angel1 was visualized using confocal microscopy. (**B**) Immunofluorescence staining was performed on HeLa S3 cells with the anti-Angel1 and Alexa 488-conjugated anti-rabbit (green) and an anti-eIF4E-specific polyclonal Alexa 555-conjugated antibody (red). Co-localization of Angel1 and eIF4E appears in yellow and is indicated by white arrows.

Because eIF4E availability for eIF4G or 4E-BP is independent of Angel1, we then explored whether Angel1 targets a specific pool of eIF4E. The cellular localizations of Angel1 and eIF4E proteins were examined (Figure 5). Indirect immunofluorescence with anti-Angel1 antibodies showed that Angel1 predominantly localizes in the cytoplasmic perinuclear area of HeLa S3 cells (Figure 5A). These results are in agreement with the localization of the overexpressed Angel1 protein (Figure 3C). As expected, no green fluorescence staining was seen after shutting down the expression of Angel1 with shRNA (Figure 5A, Supplementary Figure S5A and B). Double-labeling experiments using the anti-Angel1 and the anti-eIF4E antibodies were performed (Figure 5B). As previously demonstrated (34), eIF4E is also localized predominantly in the perinuclear area. However, strict co-localization of the two proteins was restricted to small particles as revealed by higher resolution examination (Figure 5B, lower right panel), thus confirming the idea that Angel1 targets only a fraction of eIF4E in the cell. Furthermore, the extinction of Angel1 expression did not affect the cellular localization of eIF4E (Supplementary Figure S5B), suggesting that eIF4E localization is independent of Angel1.

### Angel1 is mainly distributed in the endoplasmic reticulum and Golgi apparatus

**Figure 6.**
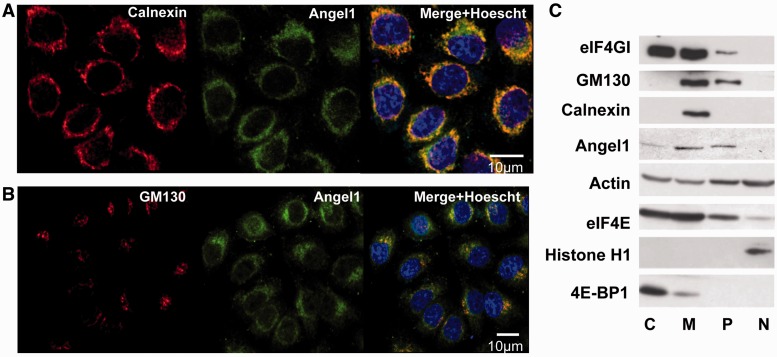
Angel1 is co-distributed with the ER and the Golgi apparatus. Co-localization of Angel1 and Calnexin (**A**), or GM130 (**B**) was determined by indirect immunofluorescence with respectively anti-Angel1 and Alexa 488-conjugated anti-rabbit (green), anti-Calnexin and Cy3-conjugated anti-mouse (red) or anti-GM130 and Cy3-conjugated anti-mouse antibodies (red). Subcellular localization of Angel1, Calnexin and GM130 were visualized using confocal microscopy. Co-localization between Angel1 and Calnexin (A), and Angel1 and GM130 (B) appears in yellow on the merged images (right panels). (**C**) Angel1 co-fractionated with Golgi and ER elements. HeLa S3 cells underwent subcellular fractionation. Nuclear (N), microsomal (M), perinuclear (P) and cytosolic fractions (C) were analyzed by western blotting with the indicated antibodies.

To gain insight into the perinuclear localization of Angel1, we then investigated whether it is distributed in organelles that are usually found at the periphery of the nucleus. Angel1 localization was compared with that of Calnexin, an endoplasmic reticulum (ER) transmembrane protein acting as a chaperone in protein folding (35) and interacting directly with ribosomes (36). A double-label immunofluorescence experiment was performed with anti-Calnexin and anti-Angel1 antibodies (Figure 6A). Angel1 co-localized primarily with the ER in these cells. A double-label immunofluorescence experiment was then performed with the anti-Angel1 and an anti-GM130 antibody, targeting a constituent of the cis-Golgi matrix facilitating ER-Golgi transport (37). As shown in Figure 6B, the higher fluorescence intensity for Angel1 and GM130 were significantly polarized on the same side of the nucleus and showed an incomplete overlap, with Angel1 displaying a wider overall distribution than the Golgi apparatus. We then addressed the specific localization of Angel1 by subcellular fractionation (Figure 6C). Western blotting was used to probe for Calnexin (ER marker), GM130 (Golgi Marker), Histone H1 (nucleus marker), eIF4E, eIF4GI, 4E-BP1 and Angel1 (Figure 6C). Angel1 was mostly present in the perinuclear (P) and microsomal (M) fractions, containing the ER and Golgi markers. We also found traces of Angel1 in cytosolic and nuclear fractions (C & N). eIF4E and eIF4GI were enriched in the cytoplasm and microsomal fractions (C & M), whereas 4E-BP1 was primarily located in the cytosol (C). Thus, Angel1 appears primarily associated with the ER.

## DISCUSSION

We identified Angel1 as a novel 4E-IP with a new strategy based on a structural and *in silico* analysis using phylogeny as a selective filter. We demonstrated the physiological interaction between Angel1 and eIF4E (Figure 2). Given that the interaction occurs through an eIF4E-binding motif similar to those found in most 4E-IPs, and that recombinant Angel1 competes with eIF4G to bind to eIF4E (Figure 2D), we infer that Angel1 binds to the interaction site located on the dorsal surface of eIF4E. The eIF4E-binding motif of Angel1 appears in the vertebrate lineage by the acquisition of an extra exon of 111 nucleotides encoding 37 amino acids (Supplementary Figure S6). We did not find the origin of this exon, suggesting that it appeared by gene transfer from a non-sequenced organism or by generation of a completely new sequence in the *angel1* gene.

We demonstrated that the binding of Angel1 to eIF4E is not sensitive to the mTOR pathway and is not affected by an increase in the eIF4E/4E-BP association (Figure 4), suggesting that 4E-BP and Angel1 do not interact with the same fraction of eIF4E in cells. Interestingly, cell fractionation showed that Angel1 and 4E-BP are not co-distributed in cells, with 4E-BP co-fractionating with cytosolic fractions while Angel1 co-distributes with the ER and Golgi elements (Figure 6C). Consequently, the association between Angel1 and eIF4E seems to occur in a 4E-BP-free cellular area, providing an explanation for the binding of Angel1 to eIF4E following PP242 treatment (Figure 4).

In the cell fractionation experiment, Angel1 co-distributed with a pool of eIF4GI and eIF4E (Figure 6C). Distribution of eIF4GI and eIF4E in microsomal and perinuclear fractions is in accordance with studies by Willet *et al.* (34,38), which show that a sub-fraction of initiation factors is associated with cellular structures and has perinuclear localization in fibroblasts (34). More importantly, a fraction of eIF4E and eIF4GI is associated with the Golgi apparatus, and some of these factors co-localize with active sites of translation, while 4E-BP is localized in the cytosol (38). Our immunofluorescence experiments revealed that Angel1 has a specific perinuclear localization, strongly associated with the ER and the Golgi apparatus (Figures 5 and 6), thereby corroborating the distribution of Angel1 in cell fractionation (Figure 6C). Moreover, Angel1 partially co-localized with eIF4E in specific particles in the perinuclear area (Figure 5B), suggesting that Angel1 interacts with a specific pool of eIF4E associated with the ER or the Golgi apparatus. Using known amounts of recombinant GST-Angel1 and GST-eIF4E (39) proteins, we determined that the ratio of eIF4E:Angel1 in HeLa S3 cell extracts is 5:1 (data not shown). This value is much higher than 1:1 ratio observed for eIF4E:4E-BP (40) and the 2:1 ratio observed for eIF4E:eIF4GI (41). We therefore conclude that Angel1 is not broadly distributed in the cell, but rather is concentrated in the endoplasmic reticulum and/or Golgi apparatus.

In eukaryotic cells, mRNAs are not homogeneously distributed in the cytoplasm. High-throughput technologies have recently uncovered that subcellular targeting of mRNAs is a widespread phenomenon (42,43). Interestingly, a substantial proportion of mRNAs are localized in the ER independently of the classical signal sequence/SRP pathway (44). Our polysome analysis of HeLa S3 cells showed that Angel1 is not associated with polysomes (Supplementary Figure S7B) and may be involved in large RNP complexes. Co-localization of eIF4E with Angel1 in perinuclear particles, as well as the co-staining of Angel1 with the Golgi apparatus and the ER, suggests that Angel1 may be involved in the regulation of specific eIF4E-bound mRNAs localized in the ER and Golgi compartments. Because these mRNAs may represent a small proportion of the total cellular mRNAs, it is not surprising that Angel1 overexpression or knockdown did not drastically affect general translation activity (Figure 3 and Supplementary Figure S5C). In accordance with the localization of Angel1, overexpressed HA-Angel1 also displayed a perinuclear pattern (Figure 3C).

Notably, we showed that, by adding recombinant Angel1 to a cell extract, Angel1 is able to compete *in vitro* with eIF4G for binding with eIF4E (Figure 2D). However we failed to observe this competition *in vivo* after HA-Angel1 overexpression (Figure 3). This discrepancy may be due to different Angel1:eIF4G ratios to eIF4E. In that case, Angel1 may compete with eIF4G only in cellular areas where this ratio is high enough to repress translation of specific localized mRNAs. Nevertheless, we cannot exclude the possibility that Angel1/eIF4E and eIF4G/eIF4E complexes may be independent and co-exist in the cell. In that case, Angel1/eIF4E may serve a more specific—still unknown—function related to the ER.

CCR4 proteins belong to the endonuclease-exonuclease-phosphatase (EEP) group and display a Mg^2+^-dependent 3′-5′ deadenylase activity that functions in the first step of the degradation of poly(A) mRNAs (45). The C-terminal domain of Angel1 displays significant homology with the conserved EEP domain carrying the deadenylase function of CCR4 (30,46). Although exonuclease 3′-5′ activity has been shown in Angel orthologs in yeast (47,48), we failed to detect any specific nuclease activity of the recombinant GST-Angel1 protein, consistent with previous observation for affinity-purified Angel1 from human cells (42). However, Angel1 has been shown to interact with CAF1B (49), which has deadenylase activity (50), suggesting that Angel1 may recruit CAF1B in eIF4E complexes. In our model, Angel1 would have two functions for regulating specific mRNAs and bringing them into translationally silenced particles. Impeding eIF4G–eIF4E interaction locally may inhibit translation, and CAF1B recruitment would lead to deadenylation of the mRNA. Specific mRNAs, following export from the nuclear pore complex, reside in association with the ER membrane in translationally silenced mRNPs, sequestered by Angel1 that interacts with eIF4E. Taken together, our results provide clear evidence that Angel1 is a new interacting partner of eIF4E and opens new perspectives for understanding the regulation of compartmentalized protein expression and eIF4E functions.

## SUPPLEMENTARY DATA

Supplementary Data are available at NAR Online: Supplementary Figures 1–7, Supplementary Methods and Supplementary References [51–56].

## FUNDING

Ligue Nationale Contre le Cancer, the Fondation ARC pour la Recherche sur le Cancer, Région Bretagne and Conseil Général du Finistère; Fondation de France and Fondation pour la Recherche Médicale (FRM) post-doctoral fellowships (to Y.M.); INSERM-Université Paul Sabatier and La Ligue Contre le Cancer (“Comités de Hautes-Pyrénées et de Lot et- Garonne” and “Equipes Labellisées” programs) (to S.P.). Funding for open access charge: CNRS.

*Conflict of interest statement.* None declared.

## Supplementary Material

Supplementary Data
