# Supplementary material for: Tracking a refined eIF4E-binding motif reveals Angel1 as a new partner of eIF4E
Source: Nucleic Acids Res. 2013 Jun 27;41(16):7783–92. doi: 10.1093/nar/gkt569 (PMC3763552; doi:10.1093/nar/gkt569)
Supplement: Supplementary Data [file supp_41_16_7783__index.html]

Tracking a refined eIF4E-binding motif reveals Angel1 as a new partner of eIF4E — Tracking a refined eIF4E-binding motif reveals Angel1 as a new partner of eIF4E — Supplementary Data 

# Tracking a refined eIF4E-binding motif reveals Angel1 as a new partner of eIF4E

## 

files

**Files in this Data Supplement:**

- Supplementary Data - pdf file
