# Supplementary material for: Tracking a refined eIF4E-binding motif reveals Angel1 as a new partner of eIF4E
Source: Nucleic Acids Res. 2013 Jun 27;41(16):7783–92. doi: 10.1093/nar/gkt569 (PMC3763552; doi:10.1093/nar/gkt569)
Supplement: Supplementary Data [file supp_gkt569_Gosselin_supp_material.pdf]

Protein name	eIF4E binding motif test
BTB/POZ domain containing protein 9	-
Virus coat protein	-
Peptidyl-prolyl cis-trans isomerase activity	ND
Cellular retinaldehyde-binding protein-like	ND
Tumor suppressor candidate 4/NPRL2/G21	-
-Hypothetical protein KIAA0355	-
Calcium channel, voltage-dependent, alpha 1E subunit	-
Activated T-cell marker CD109	ND
KCCR13L	ND
ARP10	ND
SLC39A14	ND
myosin heavy chain	ND
Reticulocalbin 2	ND
Angel homolog 1	+
eIF4G I	+
eIF4G II	ND
4E-BP1	+
4E-BP2	ND
4E-BP3	ND

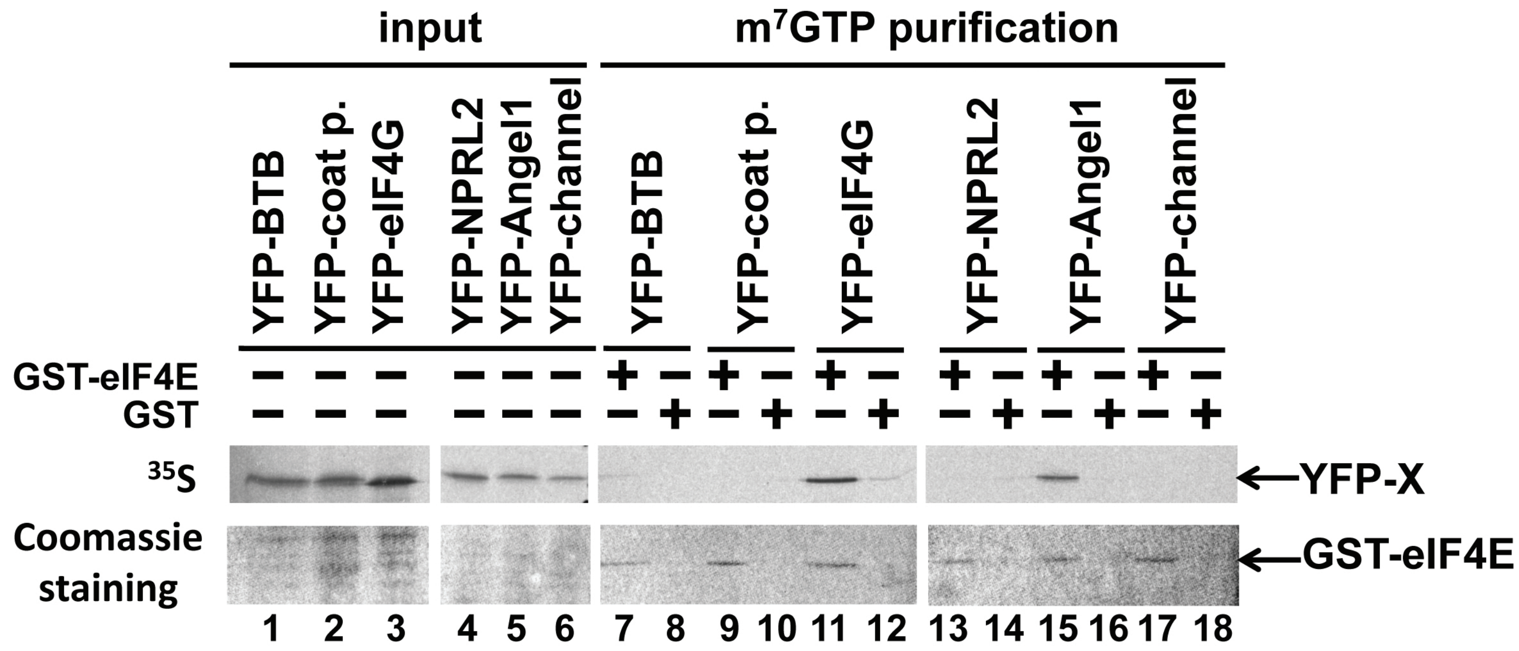
input
m7GTP purification
YFP-BTB
YFP-coat p.
YFP-eIF4G
YFP-NPRL2
YFP-Angel1
YFP-channel
GST-eIF4E
GST
35S
Coomassie staining
YFP-X
GST-eIF4E
1 2 3 4 5 6 7 8 9 10 11 12 13 14 15 16 17 18

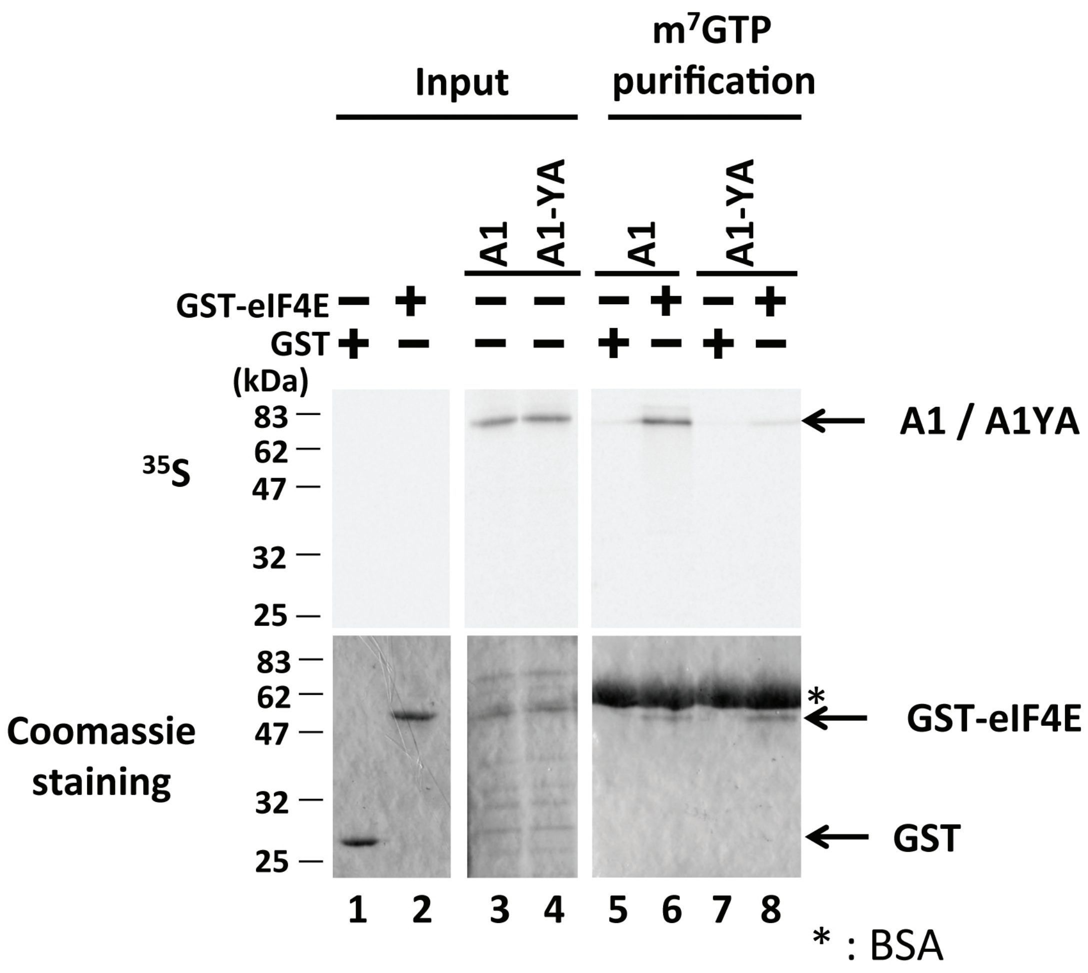

m7GTP purification
Input
A1
A1-YA
A1
A1-YA
GST-eIF4E
GST
(kDa)
83
62
47
32
25
35S
A1 / A1YA
83
62
47
32
25
Coomassie staining
*
GST-eIF4E
GST
1 2 3 4 5 6 7 8
* : BSA

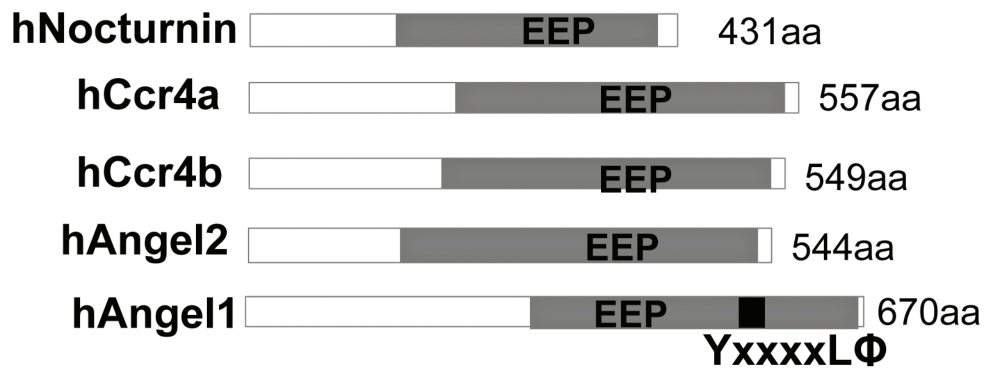
hNocturnin
EEP
431aa
hCcr4a
EEP
557aa
hCcr4b
EEP
549aa
hAngel2
EEP
544aa
hAngel1
EEP
670aa
YxxxxLΦ

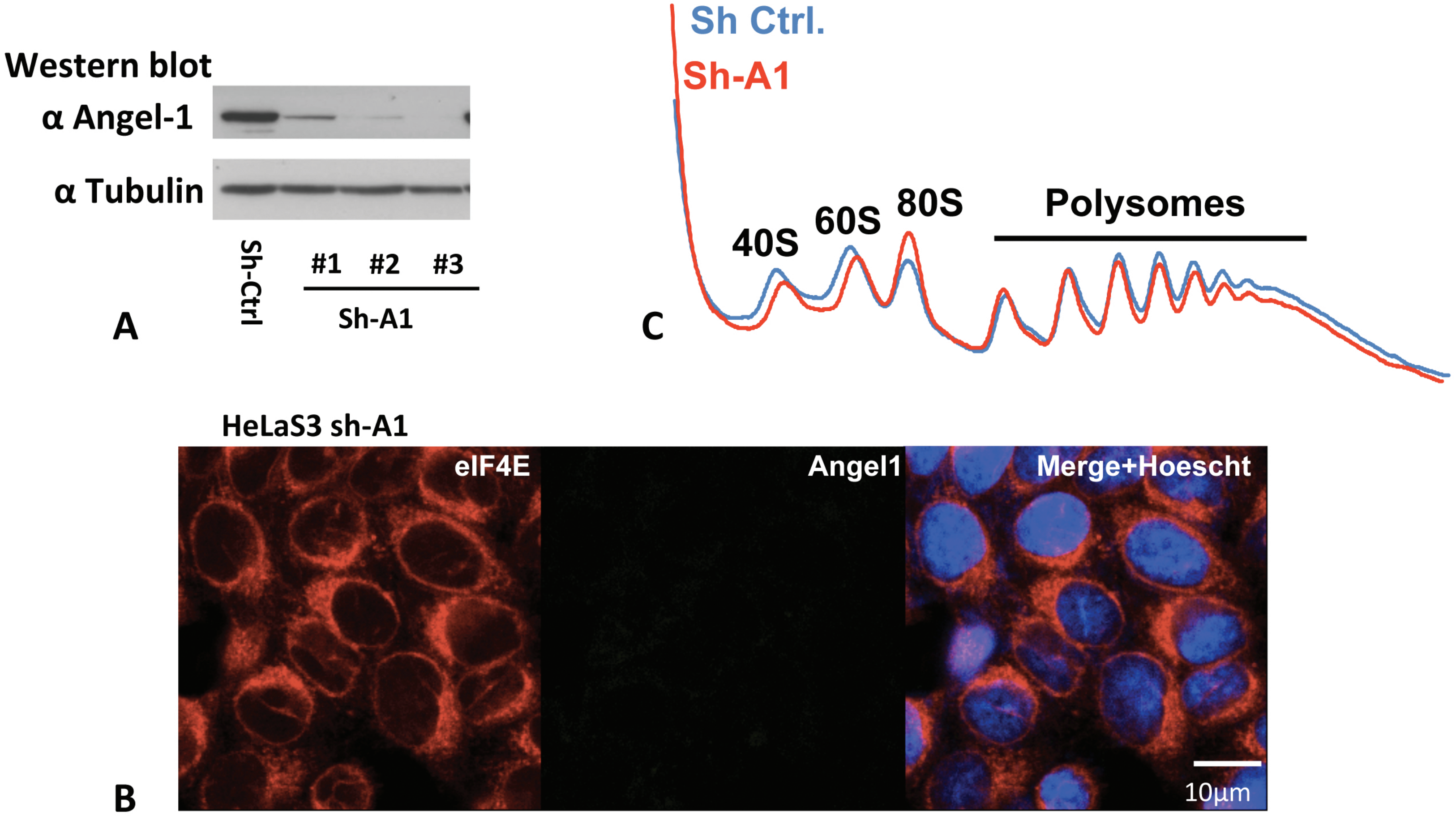

Western blot
α Angel-1
α Tubulin
Sh-Ctrl
#1
#2
#3
Sh-A1
A
Sh Ctrl.
Sh-A1
40S
60S
80S
Polysomes
C
HeLaS3 sh-A1
eIF4E
Angel1
Merge+Hoescht
10µm
B

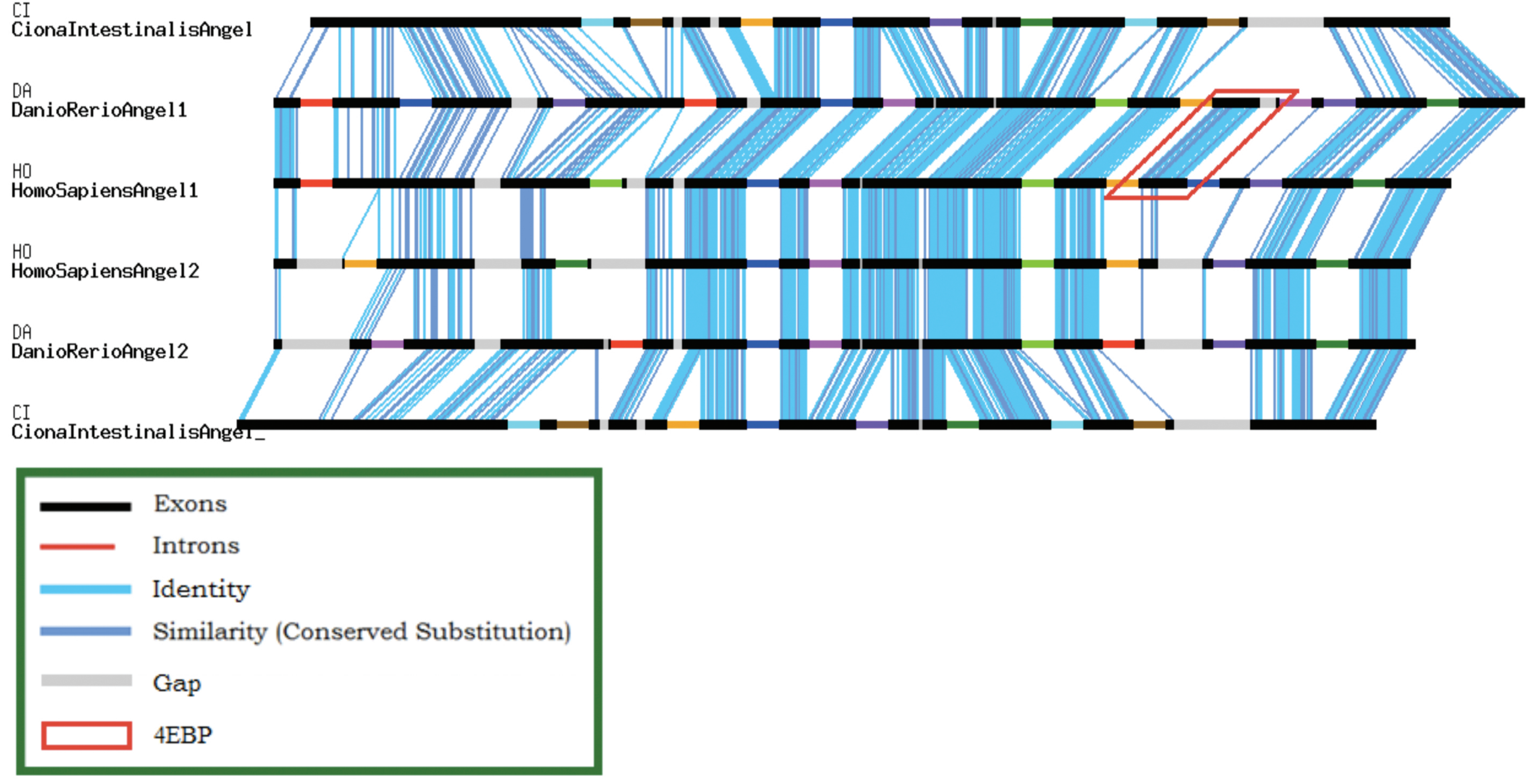
CI
CionaIntestinalisAngel
DA
DanioRerioAngel1
HO
HomoSapiensAngel1
HO
HomoSapiensAngel2
DA
DanioRerioAngel2
CI
CionaIntestinalisAngel_
Exons
Introns
Identity
Similarity (Conserved Substitution)
Gap
4EBP

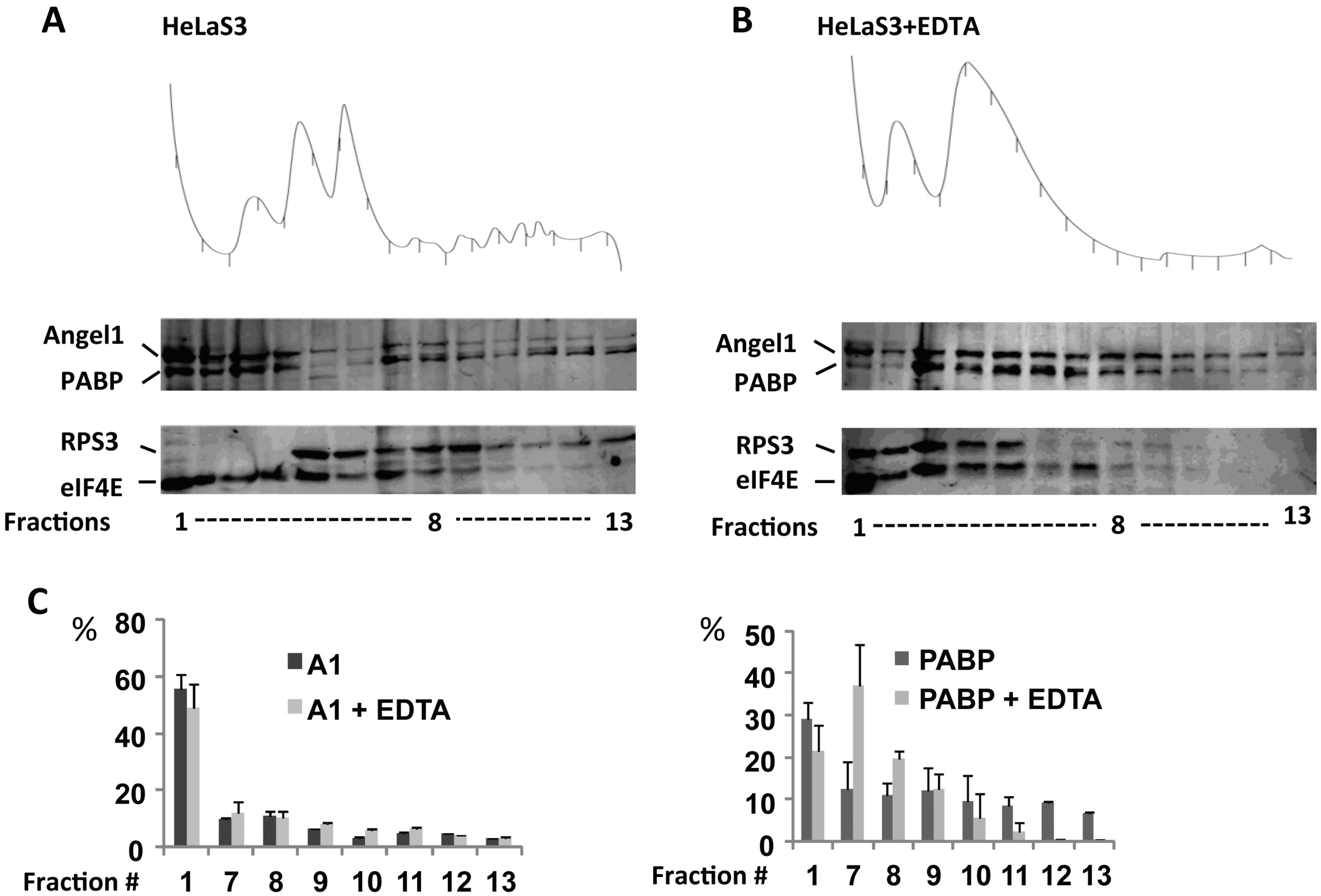

A
HeLaS3
Angel1
PABP
RPS3
eIF4E
Fractions 1 8 13
B
HeLaS3+EDTA
Angel1
PABP
RPS3
eIF4E
Fractions 1 8 13
C
%
80
60
40
20
0
A1
A1 + EDTA
Fraction #
1 7 8 9 10 11 12 13
%
50
40
30
20
10
0
PABP
PABP + EDTA
Fraction #
1 7 8 9 10 11 12 13

SUPPLEMENTARY FIGURE LEGENDS

Fig.S1: Table of the 19 groups of proteins selected to contain an eIF4E-binding motif after the in silico screening.

Left column : proteins name. Right column: result of the biochemical test that validate the interaction of these motifs with eIF4E. (+) is indicated when the interaction was observed, (-) when no interaction was observed, and ND (non determined) when the interaction was not tested.

Fig.S2: The putative eIF4E-interacting domain found in Angel1 interacts with eIF4E *in vitro*.

^{35}S-labeled putative eIF4E-interacting motifs fused to YFP (input) were produced in rabbit reticulocyte lysate and analyzed using chromatography on a m^{7}GTP column pre-loaded with GST-eIF4E or GST as control. After washing, proteins were eluted with laemmli buffer and analyzed by SDS-PAGE. Coomassie staining was used to visualize GST-eIF4E (lower panel) and Phosphorimager analysis was used to visualize ^{35}S-radiolabeled fusion proteins (upper panel).

Fig.S3: Recombinant Angel1 binds to eIF4E *in vitro*.

^{35}S-labeled wild-type (A1) or mutated (A1-YA, see text for details) Angel1 were produced in rabbit reticulocyte lysate and analysed for eIF4E binding as described in Fig. S2.

Fig.S4: Angel1 is a member of the CCR4 family

Schematic of the relative positions of eIF4E-binding site YxxxxLΦ (black box) and endo-exonuclease phosphatase (EEP) domain (gray box) in human hAngel1, hAngel2, hCCR4a and hCCR4b, and hNocturnin amino acid sequences. Amino-acid (aa) lengths of proteins are indicated.

Fig.S5 : Angel1 knock-down did not affect polysome profile of cultured cells.

A. HeLa S3 cells were transfected with pLKO vectors expressing shRNA against Angel1 (Sh-A1 #1,2,3) or Sh control (ctrl) (scramble sequence). Cells were selected for 1 week with 4 µg/mL puromycin. Expression of Angel1 in the various cell lines was analyzed by Western blotting with anti-Angel1 antibody (Sigma)**.** Anti-tubulin was used as a loading control. **B.** Immunofluorescence staining was performed on HeLaS3 cells, expressing sh-A1 #2, with anti-Angel1 and Alexa 488-conjugated (green) anti-rabbit antibodies and polyclonal anti-eIF4E Alexa 555-conjugated antibody (red). Subcellular localization of Angel1 and eIF4E were visualized using confocal microscopy. **C.** HeLa S3 cells expressing either control shRNA (sh-Ctrl, blue) or shRNA targeting Angel1 (sh-A1 #2, red) were subjected to polysomal analysis onto linear 10–40% sucrose gradient as described (52). Profiles were obtained by recording absorbance at 254 nm. Polysomes, monosome (80S), 40S and 60S ribosomal subunits are indicated. Sh-A1 expressing cell lines #1 and #3 displayed similar results for B and C.

Fig.S6: Angel's family history, an extra exon containing the eIF4E binding site appears during evolution.

Gene exon/intron organization was analyzed using GECA (53) a Perl pipeline which align exon/intron structures (MAFFT, 54) and detect common introns and similarities between sequences (CIWOG, 55). Common introns are represented with the same color. Exons (in black) are up to scale while Introns are of fixed size, gaps are in grey. The extra exon containing the eIF4E binding site is framed in red.

Fig. S7: Angel1 is involved in non-polysomal large complexes.

Angel1 associates with high-molecular-weight fractions, independently of EDTA. **A.** HeLaS3 cell extract was processed as indicated in supplementary methods. Fractions from 1 to 13 were run on a 10% SDS–PAGE gel and analyzed by immunoblotting with the indicated antibodies. Fractions 1, 2, and 3 were diluted 1:3 due to the high concentration of proteins. RPS3 and PABP were used as profile

controls. Angel1 is mostly found in the light fractions (fractions 1-3), but is also present in small amounts in the polysomal fractions (fractions 8-13) **B.** HeLa S3 cell extract was pre-treated with 50 mM EDTA and processed as described in A. Removal of Mg^{2+} ions by the addition of EDTA led to the dissociation of 80S ribosomes and polysomes into partially disassembled and unfolded ribosomal subunits and the release of mRNA (56). In EDTA-treated extracts, even though we observed a marked increase in the abundance of free ribosomal subunits and the disappearance of 80S monosomes and polyribosomes, Angel1 was still observed in the heavy fractions (fractions 8-13) **C.** Amounts of Angel1 and PABP in the gradient fractions 1, and 7 to 13 with or without EDTA quantified by densitometric analysis of Western blots using Imagequant software. The percentage of proteins per fraction is calculated against the total amount of proteins, determined by adding fractions 1 and 8 to 13. Error bars were established from two different experiments. While PABP shifted to the polysomes gradient upper-most fractions upon EDTA treatment (fractions 1-8), Angel1 displayed the same distribution whether extracts were treated with or without EDTA.

SUPPLEMENTARY REFERENCES

52. Mamane, Y., Petroulakis, E., Martineau, Y., Sato, T.A., Larsson, O., Rajasekhar, V.K. and Sonenberg, N. (2007) Epigenetic activation of a subset of mRNAs by eIF4E explains its effects on cell proliferation. *PLoS One*, **2**, e242.
53. Fawal, N., Savelli, B., Dunand, C. and Mathe, C. (2012) GECA: a fast tool for gene evolution and conservation analysis in eukaryotic protein families. *Bioinformatics*, **28**, 1398-1399.
54. Katoh, K., Misawa, K., Kuma, K. and Miyata, T. (2002) MAFFT: a novel method for rapid multiple sequence alignment based on fast Fourier transform. *Nucleic acids research*, **30**, 3059-3066.
55. Wilkerson, M.D., Ru, Y. and Brendel, V.P. (2009) Common introns within orthologous genes: software and application to plants. *Briefings in bioinformatics*, **10**, 631-644.
56. Nolan, R.D. and Arnstein, H.R. (1969) The dissociation of rabbit reticulocyte ribosomes into subparticles active in protein synthesis. *European journal of biochemistry / FEBS*, **10**, 96-101.
57. Parsyan, A., Shahbazian, D., Martineau, Y., Petroulakis, E., Alain, T., Larsson, O., Mathonnet, G., Tettweiler, G., Hellen, C.U., Pestova, T.V. *et al.* (2009) The helicase protein DHX29 promotes translation initiation, cell proliferation, and tumorigenesis. *Proceedings of the National Academy of Sciences of the United States of America*, **106**, 22217-22222.

SUPPLEMENTARY METHODS

Constructions: Angel1 cDNA was amplified with the primers A15' (CGGCCATGATCGCGTCGTGC) and A13' (TCATGGGGCGGTGACTTCCATCCC) and Pfu polymerase (Promega). PCR products purified from agarose gels were subcloned into pcr2.1 topo using the Topo TA cloning kit (Invitrogen) for DNA sequencing. The Angel1 open reading frame was then amplified from a 5' *Xba*I site and HA tag to a 3' *Not*I site using primers XbaHAA1(ATATAATCTAGAATGTACCCCTATGATGTGCCCGATTATGCCATGATCGCGTCGTGCTTGTG) and A1NotI (ATAATGCGGCCGCCCCTGGGAGCCCTGTCATGGG) and then subcloned into the *Nhe*I/*Not*I sites of a pCI-neo mammalian expression vector (Promega).

To express GST-Angel1 (A1), the PCR-generated DNA fragment containing the targeted coding region of Angel1 was cloned into the *Eco*RI and *Bam*HI sites of a pGEX-4T-2 plasmid (Amersham Pharmacia Biotech). A single point mutation of the residue Tyr_{506} (Angel1YA (A1YA)) was created in the pcr2.1, pCI-neo and pGEX-4T-2 constructs using the Quick-change^{TM}site-directed mutagenesis kit (Stratagene) following the manufacturer's instructions. The following two primers were used for mutagenesis: $Glu_{298\text{-}Ala}$F(AGATCAGAGAGACGCAAGGCTGGCCGAGACTTCCTGCTACG) and

$Glu_{298\text{-}Ala}$R(CGTAGCAGGAAGTCTCGGCCAGCCTTGCGTCTCTCTGATCT). The pcr2.1 constructs were used to produce recombinant Angel1 in rabbit reticulocyte lysate.

Antibodies: The following antibodies were used: anti-eIF4GI and anti-4E-BP2 obtained from Dr. N. Sonenberg (McGill University, Montreal, Canada); anti-4E-BP1, RPS3, PABP and calnexin (Cell Signaling Technology); anti-eIF3b, GAPDH, Histone H1 and GST (Santa Cruz Biotechnology, Inc); anti-β-Tubulin, β-Actin, HA-7 and Angel-1 (Sigma-Aldrich); anti-eIF4E and anti-GM130 (Transduction Laboratories); anti-HA (Roche Boehringer); anti-GFP (abcam); anti-rabbit, anti-rat and anti-mouse secondary antibodies for Western blots (Dako SA). Rabbit polyclonal anti-eIF4E antibody conjugated with rhodamin for immunofluorescence was a king gift from S. Morley (University of Sussex, Brighton, UK) (40). Secondary antibodies for immunofluorescence including goat anti-rabbit Alexa 555, goat anti-rabbit Alexa 488, goat anti-mouse Cy3, goat anti-mouse Alexa 488 and anti-rat TRITC T4280 (all from Invitrogen). Figure 1A was performed with conformation-specific rabbit secondary antibody (Cell Signaling Technology).

Cell culture: HeLaS3, and HEK293 cells were obtained from the American Tissue Culture Collection and maintained in Dulbecco's minimum essential medium (DMEM, Lonza) supplemented with 10% fetal bovine serum (FBS, Lonza) and 5 U/mL penicillin-streptomycin (Lonza) in 5% CO_2. Constitutive silencing of Angel1 was obtained using pLKO vectors (Sigma-Aldrich) as previously described (52). shRNA vector accession numbers are as follows: Angel1 #1 (TRCN0000127505), Angel1 #2 (TRCN0000128671), Angel1 #3 (TRCN0000128796) and non-target shRNA control SHC002. For PP242 (Sigma-Aldrich) treatments, cells were incubated 1 h with 2.5 μM PP242 before harvesting.

Phylogeny: Sequences used were XP_001637908.1 (N.vectensis), NP_477204.1 (D.Melagogaster), SPU_021986 (S.purpuratus), XM_002128787.1 (C.intestinalis), NP_001025131.1 (D.rerio), NP_001089542.1 (X.laevis), NP_001026218.1 (G.gallus), NP_067396.3 (M.musculus), NP_653168.2 (H.sapiens), XP_698034.2 (D.rerio), NP_001016236.1 (X.laevis), NP_001026376.1 (G.gallus), NP_653107.2 (M.musculus), NP_056120.2 (H.sapiens).

HA-immunoprecipitation assay: HeLaS3 cells were transiently transfected with pCI-neo constructs using Lipofectamine 2000 (Invitrogen) in OPTI-MEM (Invitrogen), according to the manufacturer's instructions. Co-immunoprecipitations were performed with ProFound™ Mammalian HA Tag IP/Co-IP Kit (Thermo Scientific) following the manufacturer's instructions.

Immunoprecipitation and m^7GTP purification: HeLaS3 cells were seeded on 150 mm plates and treated with a vehicle (DMSO) or PP242 (2.5 μM) for 1 h. Cells were then harvested as described in ref. 57. When indicated, cell extracts were treated with 200 μg/mL RNase A (Invitrogen) at 37°C for 15 min to exclude the possibility of RNA-mediated interactions. Cell extracts were transferred onto 7-methyl GTP sepharose 4B (GE Healthcare) or anti-eIF4E sepharose (Santa Cruz Biotechnology, Inc.) or protein G-sepharose beads (GE Healthcare) with indicated antibody before incubation at 4°C for 2 h. Beads were washed four times and bound proteins processed for Western blotting.

Expression, production, eIF4E-binding assay: The wild type and mutant proteins GST-A1, GST-A1YA were overexpressed in *E. coli* (Rosetta (BL21), Novagen) and purified on a glutathione sepharose 4B column (Amersham Pharmacia Biotech) according to the manufacturer's instructions. Proteins were eluted in buffer EB (50 mM Tris- HCl, 10 mM reduced glutathione (Sigma-Aldrich), pH 8) and were run on a 10% SDS polyacrylamide gel to analyze their quality. Blue coomassie gel staining was used to quantify purified proteins. 1 μg of each protein was added to cell extract for 1 h at 4°C, and then incubated with 7-methyl GTP sepharose 4B beads for 2 h at 4°C. Beads were washed four times and processed for Western blotting.

Immunofluorescence: HeLaS3 cells (3.10^3) were plated on microplates CellCarrier™-96 (Perkin Elmer, 6005550) and grown for 48 h at 37°C. Cells were washed three times with PBS, fixed by 3% PFA in PBS at room temperature (RT) for 15 min and washed again. Membranes were permeabilized

with 0.5% Triton in PBS/NH_4Cl (wash buffer) at RT for 10 min. After washes, cells were incubated at RT for 1 h with 2.5% guinea pig serum before incubation with primary antibody and 2.5% guinea pig serum overnight at 4°C. Incubation with the appropriate secondary antibody was performed for 1 h at RT after washes. Finally, cells were stained for 10 min with 1 µg/mL Hoechst 33342 (Sigma-Aldrich) in wash buffer and washed twice. Images were collected on a confocal Leica SP5 microscope using a 40X or 63X oil objective.
